# Tissue Proteomic Analysis Identifies Mechanisms and Stages of Immunopathology in Fatal COVID-19

**DOI:** 10.1165/rcmb.2021-0358OC

**Published:** 2021-10-28

**Authors:** Clark D. Russell, Asta Valanciute, Naomi N. Gachanja, Jillian Stephen, Rebekah Penrice-Randal, Stuart D. Armstrong, Sara Clohisey, Bo Wang, Wael Al Qsous, William A. Wallace, Gabriel C. Oniscu, Jo Stevens, David J. Harrison, Kevin Dhaliwal, Julian A. Hiscox, J. Kenneth Baillie, Ahsan R. Akram, David A. Dorward, Christopher D. Lucas

**Affiliations:** ^1^ University of Edinburgh Centre for Inflammation Research, Queen’s Medical Research Institute, Edinburgh BioQuarter, Edinburgh, United Kingdom;; ^2^ Roslin Institute, University of Edinburgh, Easter Bush Campus, Midlothian, United Kingdom;; ^3^ Institute of Infection, Veterinary and Ecological Sciences, University of Liverpool, Liverpool, United Kingdom;; ^4^ Department of Pathology, Western General Hospital, Edinburgh, United Kingdom;; ^5^ Department of Pathology,; ^6^ Edinburgh Transplant Centre,; ^8^ Department of Respiratory Medicine, and; ^11^ Intensive Care Unit, Royal Infirmary of Edinburgh, Edinburgh, United Kingdom;; ^7^ School of Medicine, University of St. Andrews, North Haugh, St. Andrews, United Kingdom;; ^9^ NIHR Health Protection Research Unit in Emerging and Zoonotic Infections, Liverpool, United Kingdom;; ^10^ Infectious Diseases Horizontal Technology Centre, Agency for Science, Technology, and Research, Singapore; and; ^12^ Institute for Regeneration and Repair, University of Edinburgh, Edinburgh BioQuarter, Edinburgh, United Kingdom

**Keywords:** COVID-19, lung, inflammation, macrophages, proteomics

## Abstract

Immunopathology occurs in the lung and spleen in fatal coronavirus disease (COVID-19), involving monocytes/macrophages and plasma cells. Antiinflammatory therapy reduces mortality, but additional therapeutic targets are required. We aimed to gain mechanistic insight into COVID-19 immunopathology by targeted proteomic analysis of pulmonary and splenic tissues. Lung parenchymal and splenic tissue was obtained from 13 postmortem examinations of patients with fatal COVID-19. Control tissue was obtained from cancer resection samples (lung) and deceased organ donors (spleen). Protein was extracted from tissue by phenol extraction. Olink multiplex immunoassay panels were used for protein detection and quantification. Proteins with increased abundance in the lung included MCP-3, antiviral TRIM21, and prothrombotic TYMP. OSM and EN-RAGE/S100A12 abundance was correlated and associated with inflammation severity. Unsupervised clustering identified “early viral” and “late inflammatory” clusters with distinct protein abundance profiles, and differences in illness duration before death and presence of viral RNA. In the spleen, lymphocyte chemotactic factors and CD8A were decreased in abundance, and proapoptotic factors were increased. B-cell receptor signaling pathway components and macrophage colony stimulating factor (CSF-1) were also increased. Additional evidence for a subset of host factors (including DDX58, OSM, TYMP, IL-18, MCP-3, and CSF-1) was provided by overlap between *1*) differential abundance in spleen and lung tissue; *2*) meta-analysis of existing datasets; and *3*) plasma proteomic data. This proteomic analysis of lung parenchymal and splenic tissue from fatal COVID-19 provides mechanistic insight into tissue antiviral responses, inflammation and disease stages, macrophage involvement, pulmonary thrombosis, splenic B-cell activation, and lymphocyte depletion.

Fatal coronavirus disease (COVID-19) is associated with pronounced inflammatory changes in the lung and reticuloendothelial system (1). Despite the widespread presence of the causative severe acute respiratory syndrome coronavirus 2 (SARS-CoV-2) virus, there is minimal histological evidence of viral cytotoxicity or substantial inflammation in other organs (1–4). Within the lung, the presence of virus is not consistently associated with the occurrence or nature of the inflammatory response. This disconnect between the presence of virus and inflammation identifies virus-independent immunopathology as a pathophysiologic feature of severe COVID-19. Pulmonary inflammation is characterized by an expansion of macrophages/monocytes in the lung parenchyma, a mononuclear cell pulmonary artery vasculitis, microthrombosis, and increased numbers of megakaryocytes (1, 5, 6). In the spleen, bone marrow, and mediastinal lymph nodes, there is a stereotyped reactive plasmacytosis and expansion of activated macrophages (1, 2). The biological mechanisms leading to and from these immunopathogenic changes are unknown.

Antiinflammatory therapy with corticosteroids substantially improves survival for patients with hypoxic respiratory failure owing to COVID-19, indicating that inflammatory processes are causal in death (7, 8). IL-6 blockade has an additive effect to the benefit of corticosteroids (9). Despite these advances, mortality remains high. An improved mechanistic understanding of inflammation in COVID-19 may help to improve upon these therapies. Studies of soluble mediators in peripheral blood have described activation of prothrombotic pathways, endothelial injury, and macrophage/monocyte chemotactic signaling in this compartment, but characterization of inflamed tissues is urgently needed (10–12). We aimed to gain mechanistic insight into fatal COVID-19 through proteomic analysis of pulmonary and splenic tissue, thereby identifying molecular targets implicated in tissue immunopathology.

Some of the results of these studies have been previously reported in the form of a preprint (https://papers.ssrn.com/sol3/papers.cfm?abstract_id=3854606).

## Methods

### COVID-19 Postmortem Examinations

Consent to undertake postmortem examinations was obtained from next of kin. Ethical approval was granted by the East of Scotland Research Ethics Service (16/ES/0084). During April–June 2020, postmortem examinations were conducted on 13 patients with premortem PCR-confirmed SARS-CoV-2 infection and radiologic evidence of pneumonitis as previously reported (1). In this study, tissue from right middle lobe of lung and the spleen was used. Postmortems were performed a mean of 19.8 hours (SD ± 19 h) after death. Formalin-fixed paraffin-embedded tissue blocks were used in histologic analyses, including semiquantitative scoring of pulmonary inflammation. Tissue samples were TRIzol treated for subsequent protein and total RNA extraction. Viral RNA was detected by multiplex PCR (ARTIC Network protocol), confirmed by Nanopore sequencing, as previously reported (1).

### Control Tissue Samples

Ethical use of control uninflamed lung tissue was approved by National Health Service Lothian Scottish Academic Health Sciences Collaboration Bioresource (REC No.: 15/ES/0094). All participants provided written informed consent prior to enrollment in the studies. Control lung tissue was obtained from archived tissue (predating the SARS-CoV-2 pandemic) obtained at the time of lung cancer resection from seven patients. Areas of macroscopically normal noncancerous lung were dissected and frozen at −80°C until use. Absence of inflammation and tumor involvement was confirmed by hematoxylin and eosin staining and analysis by a thoracic histopathologist. Control splenic tissue was obtained from the UK Quality in Organ Donation (QUOD) biobank from 12 deceased organ donors. Approval for the use of tissue was granted under QUOD ethical approval (RAP066: Understanding COVID-19 Impact).

### Protein Extraction

Protein was extracted from TRIzol-treated case and control tissues using a phenol extraction technique described in the data supplement. Total protein content was normalized prior to proteomic analysis.

### Proteomics

Identification and quantification of tissue protein extracts was performed by Olink using multiplex immunoassay panels (“Inflammation” version 3022, “Immune Response” version 3203, “Organ Damage” version 3311, and “Metabolism” version 3403; Olink). The protein targets of these panels are listed in Table E1 in the data supplement. Assay details are also reported in the data supplement.

### Analysis

Protein immunoassay measurements were normalized and log_2_ transformed to generate arbitrary normalized protein expression units. A higher value represents a higher protein abundance. Protein targets were excluded if more than 50% of results were less than the lower limit of detection (LLOD). Proteins included in the analyses are listed in Tables E2 and E3. Remaining results less than LLOD were imputed with LLOD/√2, as previously described (13). For protein targets included in both the “Inflammation” and “Immune Response” panels, correlation analysis demonstrated excellent intrasample reproducibility (Pearson *R*^2^ values of 0.92–1.0) (Figures E1C–E1G). Differential expression analysis of proteins was performed in R Studio (version 1.3.959) using the *limma*, *prcomp* and *ggplot* packages (14). A false discovery threshold of less than 0.05 was used to define differentially abundant proteins. Patient clusters were identified by network analysis using the Markov Clustering (MCL) Algorithm (Graphia, version 2.0 [15]) or hierarchical clustering using the *pheatmap* package in R Studio. The Enrichr web server was used for gene set enrichment analysis (16, 17). Additional plotting and statistical tests were performed using GraphPad Prism (version 9.0.0).

## Results

### Patients

Characteristics of the patients with fatal COVID-19, and histologic findings, are presented in Table 1. Patients had a mean age of 79.6 years (±12.7), were predominantly male (12:1 male:female), and had a mean illness duration, from onset to death, of 21.5 days (±10.4). All had hypoxic respiratory failure and radiologic evidence of pneumonitis, and four received invasive mechanical ventilation (IMV). All patients had histologic evidence of pulmonary inflammation in the same lung lobe used for proteomic analysis (right middle lobe), with diffuse alveolar damage in 9/13. Pulmonary microthrombosis was present in the right middle lobe in 4/13 patients with a premortem radiological diagnosis of pulmonary embolism in three, but in no cases was this considered to be the direct cause of death. As previously described (1), a reactive plasmacytosis was seen in the spleen in all patients, with white pulp atrophy observed in 5/13 patients. Peripheral blood lymphopenia in the sample closest to time of death was present in 10/13 patients, with a median count of 0.64 × 10^9^/L (interquartile range, 0.44–1.3; normal, >1.5). Eleven of these patients were included in a previous report (1). Control lung tissue donors had a mean age of 69.0 years (±5.0) and comprised 3:4 male:female. Spleen tissue donors had a mean age of 74.3 years (±7.2); all were male (Table E5).

**Table 1.** Characteristics of Patients with Fatal COVID-19

	*n* = *13*
Age, y	79.6 ± 12.7
Sex, male:female	12:1
Illness duration, d	21.5 ± 10.4
Clinical and radiological features	
Hypoxic respiratory failure	13 (100)
Thoracic radiology	
Pulmonary GGO	13 (100)
Pulmonary embolism	3 (23.1)
Supportive care	
Supplemental oxygen	13 (100)
Invasive mechanical ventilation	4 (30.8)
Duration (intubation to death), d	18.3 ± 7.8
Vasopressors	4 (30.8)
Renal replacement therapy	3 (23.1)
Histological findings	
Lung*	
Inflammation	13 (100)
Diffuse alveolar damage	9 (69.2)
Thrombosis	4 (30.8)
Spleen	
Reactive plasmacytosis	13 (100)
White pulp atrophy	5 (38.5)
SARS-CoV-2 RNA detected	
Spleen	5 (38.5)
Lung*	8 (61.5)

*Definition of abbreviations*: GGO = ground glass opacification; SARS-CoV-2 = severe acute respiratory syndrome coronavirus 2.

Data are presented as mean ± SD or absolute number (% of total).

* Findings in right middle lobe (same lobe used for proteomic analysis).

### Lung Parenchyma Proteomic Analysis

**Figure 1.**
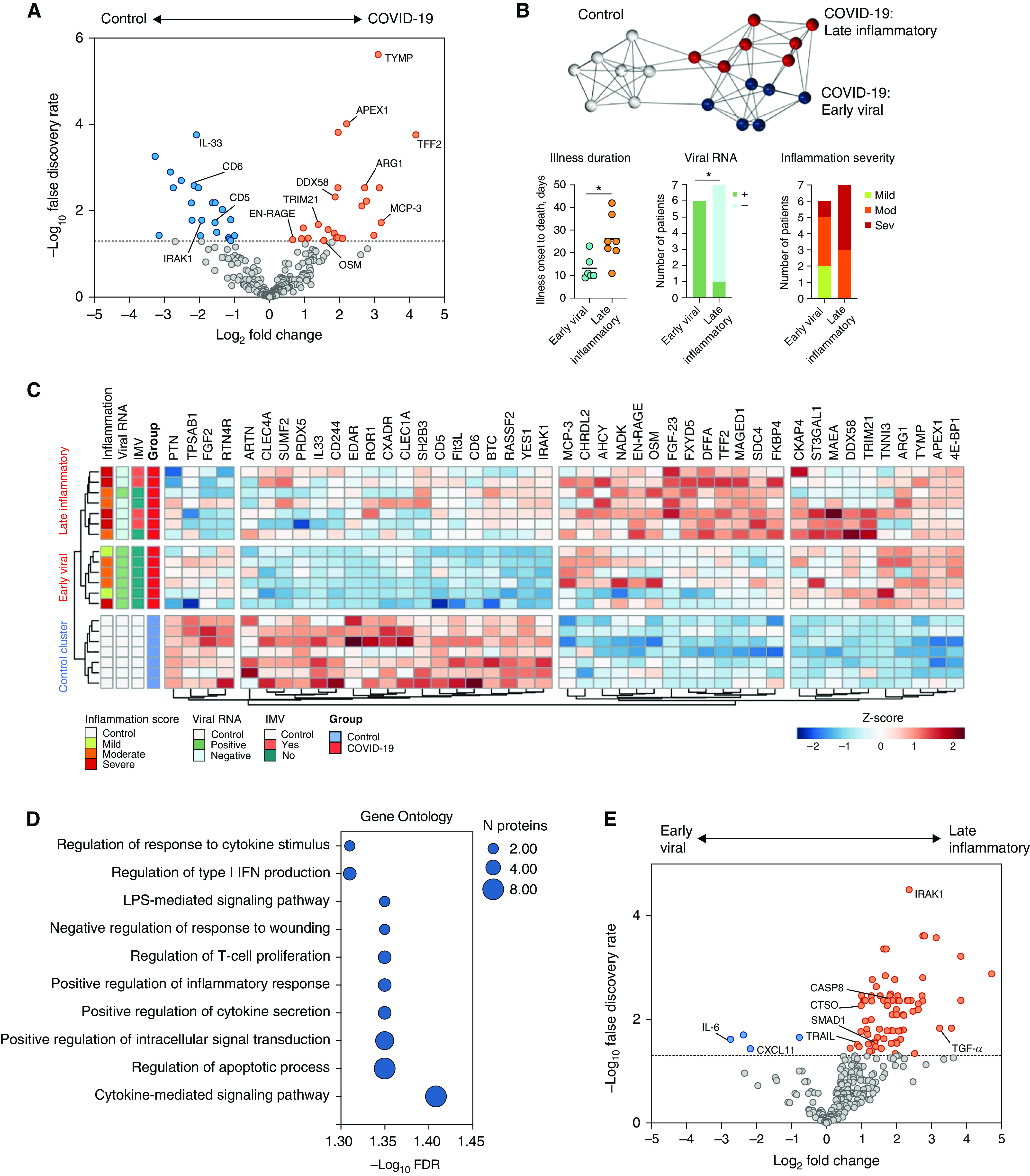
Proteomic analysis of lung parenchymal tissue in fatal coronavirus disease (COVID-19). (*A*) Differential protein abundance in patients with COVID-19 compared with control subjects. Volcano plot of log_2_ fold change difference in protein abundance. Horizontal dotted line indicates false discovery rate of 0.05. Genes are colored based on differential abundance (false discovery rate [FDR] < 0.05): increased (orange), decreased (blue), or no difference (gray). (*B*) Patient-to-patient network analysis. Lung proteomic data was used to identify three clusters of patients. Edges represent connections with a Pearson correlation value of at least 0.85. The *k*-nearest neighbors method was used for edge reduction (*k* = 5). Nodes represent patients and are colored by cluster membership, determined using the Markov clustering algorithm (granularity 2.8). Plots below the network show differences in illness duration before death, presence of viral RNA, and inflammation severity between the two COVID-19 clusters. (*C*) Clustered heatmap of differential protein abundance. Clusters of proteins (columns) and patients (rows) were determined by hierarchical clustering (reproducing the same patient clusters as the Markov clustering method in panel [*B*]) and are represented by dendrograms. Metadata relating to each patient are shown by colored annotations: histological inflammation score of lung tissue used in analysis, presence/absence of viral RNA, and receipt of invasive mechanical ventilation (IMV) prior to death. Shading of cells represents the *z*-score, computed on a protein-by-protein basis. (*D*) Gene set enrichment of differentially abundant proteins in fatal COVID-19 lung parenchyma (FDR < 0.05) in the Gene Ontology Biological Processes database (no significantly enriched KEGG or WikiPathways pathways were identified). (*E*) Differential protein abundance in the late inflammatory cluster compared with the early viral cluster. Volcano plot of log_2_ fold change difference in protein abundance. Horizontal dotted line indicates FDR of 0.05. Genes are colored based on differential abundance (FDR < 0.05): increased (orange), decreased (blue), or no difference (gray).

Principal component analysis of the pulmonary proteomic data (267 proteins) demonstrated separation of tissue samples from patients with fatal COVID-19 and patients who were not infected with SARS-CoV-2, along the second principal component (Figure E2). In comparison with normal lung tissue, 22 proteins were decreased in abundance and 23 were increased in patients with fatal COVID-19 (Figure 1A and Table E2). Gene ontology terms relating to type 1 IFN production, cytokine secretion, inflammatory response, wounding, and apoptosis were significantly enriched in the differentially abundant proteins (Figure 1D). Specific proteins of interest are labeled in Figure 1A. These results demonstrate an increased abundance of the cytosolic pattern recognition receptor protein DDX58 (also known as RIG-I) and the cytosolic antibody receptor TRIM21 in the lung of patients with COVID-19, with a positive correlation existing between these two antiviral immune components (*R*^2^ = 0.65; *P* = 0.0009) (Figure S3A). Other proteins that were increased in abundance included the monocyte/macrophage chemoattractant MCP-3 (monocyte-chemotactic protein 3), the prothrombotic platelet product TYMP (thymidine phosphorylase), the repair factor TFF2 (trefoil factor 2), and regulator of oxidative stress responses APEX1 (apurinic/apyrimidinic endoDNase 1). OSM (oncostatin M, a positive regulator of IL-6 signaling) and EN-RAGE (S100A12, a biomarker of inflammation in acute respiratory distress syndrome [ARDS]) were increased in abundance in fatal COVID-19, positively correlated (*R*^2^ = 0.67; *P* = 0.0006) and elevated independent of whether the patient had received IMV (Figure E3B). EN-RAGE and OSM were both elevated in patients with moderate–severe compared with mild pulmonary inflammation (Figure S3C-D). Proteins that were decreased in abundance in fatal COVID-19 included the epithelial damage-associated molecular pattern IL-33, IL-1 receptor-associated kinase-1 (IRAK1), and the lymphocyte receptors CD5 and CD6.

Unsupervised clustering of the lung proteomic data alone, using two distinct methods (MCL and hierarchical clustering), identified three clusters of proteomic profiles. When associated meta-data (not included in the analysis) was inspected, these represented uninfected controls and two COVID-19 clusters (Figures 1B and 1C). The two COVID-19 clusters differed based on presence of viral RNA, severity of pulmonary inflammation (histologically and based on proteomic profiles [Figure 1C]), and illness duration before death (Figure 1B). Based on these parameters, we assigned the COVID-19 clusters the designations “early viral” and “late inflammatory.” The early viral cluster all had detectable viral RNA (6/6) in the analyzed lung sample, and inflammation was mostly minor–moderate. In contrast, only 1/7 patients in the late inflammatory cluster had detectable virus and inflammation was moderate–severe. The mean duration of illness prior to death was substantially longer in this cluster: 26.1 versus 13.2 days (*P* = 0.02). Although 4/7 patients in the late inflammatory cluster had received IMV, these patients had similar protein abundance profiles to the other patients in the cluster, indicating this clustering did not simply reflect ventilator-induced lung injury. Differential expression analysis between the early viral and late inflammatory cases identified increased abundance of proteins including IRAK1, proapoptotic TRAIL, cathepsin O and caspase 8, proangiogenic SMAD1, and profibrotic TGF-α, and reduced abundance of IL-6 in the late inflammatory cluster (Figure 1E and Table E3).

### Spleen Proteomic Analysis

**Figure 2.**
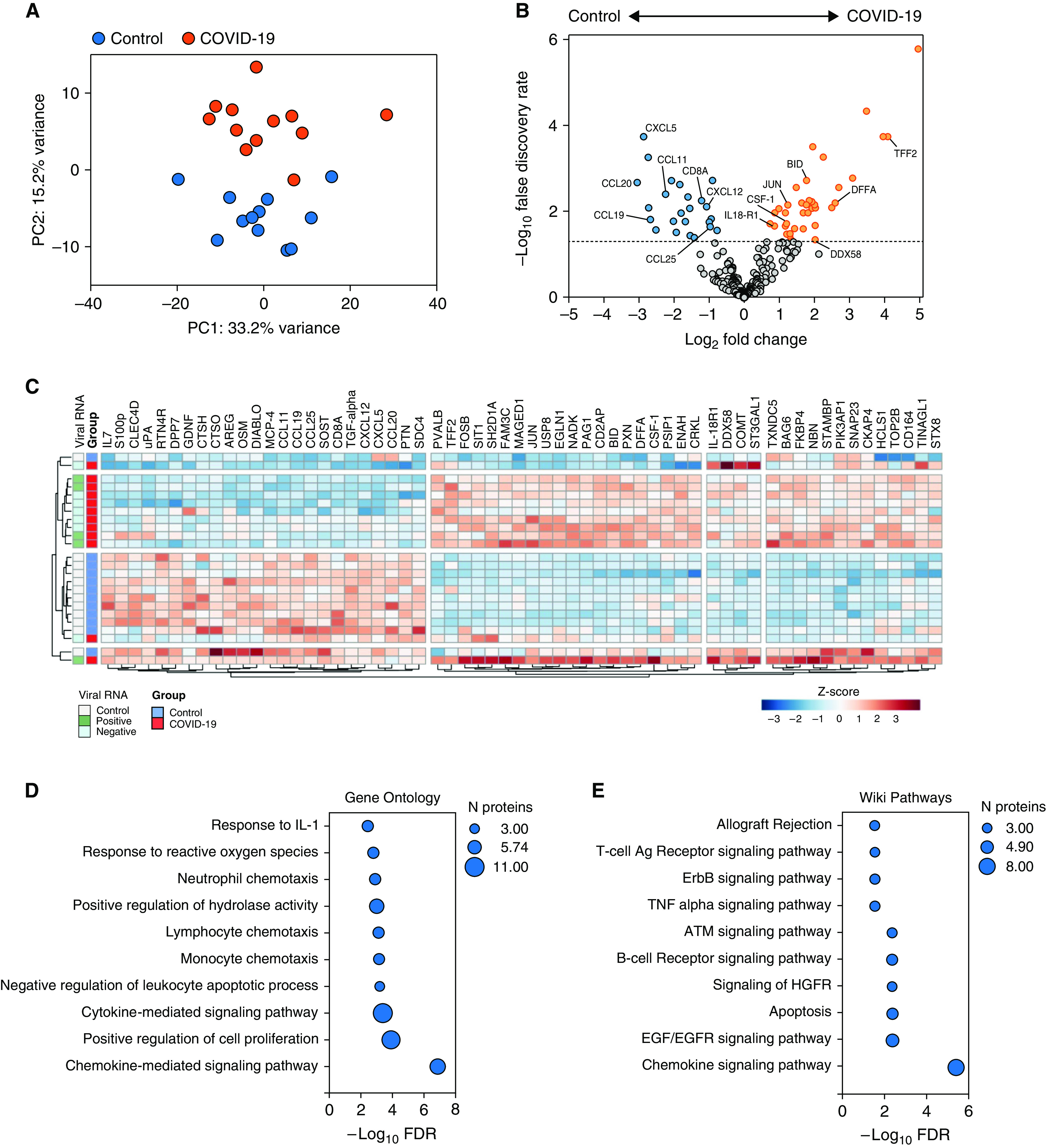
Proteomic analysis of splenic tissue in fatal COVID-19. (*A*) Principal component analysis of 316 protein concentrations in splenic tissue from patients with fatal COVID-19 and deceased organ donors (control subjects). COVID-19 samples are shown in red and control samples in blue. (*B*) Differential protein abundance. Volcano plot of log_2_ fold change difference in protein abundance between COVID-19 and control samples. Horizontal dotted line indicates FDR of 0.05. Genes are colored based on differential abundance (FDR < 0.05): increased (orange), decreased (blue), or no difference (gray). (*C*) Clustered heatmap of differential protein abundance. Clusters of proteins (columns) and patients (rows) were determined by hierarchical clustering and are represented by dendrograms. Viral presence is shown by colored annotation. Shading of cells represents the z-score, computed on a protein-by-protein basis. Gene set enrichment of differentially abundant proteins in fatal COVID-19 spleen tissue (FDR < 0.05) using the (*D*) Gene Ontology Biological Processes and (*E*) WikiPathways databases.

Principal component analysis of the proteomic data (316 proteins) demonstrated segregation of tissue samples from patients with fatal COVID-19 along the second principal component (Figure 2A and Table E4). In comparison to control spleen tissue, 24 proteins were decreased in abundance and 37 were increased in patients with fatal COVID-19 (Figures 2B and 2C). Enriched pathways and gene ontology terms in differentially abundant proteins included those relating to chemokine signaling, leukocyte chemotaxis, and apoptosis (Figures 2D and 2E). Hierarchical clustering of the proteomic data identified two major clusters broadly reflecting patients with COVID-19 and uninfected controls (Figure 2C). The presence of viral RNA in spleen tissue was not associated with any clustering of proteomic data for patients with COVID-19. A decreased abundance of a cluster of several leukocyte chemotactic factors (MCP-4, CXCL12, CCL11, CCL19, CCL25, and CCL20) was observed in the COVID-19 cases and associated with a decrease in the cytotoxic T-cell marker CD8A. Increased abundance of the proapoptotic factors DNA fragmentation factor subunit α (DFFA), Bcl2 family member BID (BH3 interacting-domain), and cathepsin O and H (overlapping with “Apoptosis” KEGG and WikiPathway pathways) was also observed. The proteins overlapping with the enriched “B-cell Receptor Signaling Pathway (WP23),” JUN, HCLS1, PIK3AP1, and CRKL, were all increased in abundance in the COVID-19 spleen tissue, in addition to IL-18R1. Macrophage CSF-1 was also increased in abundance in COVID-19 splenic tissue.

### Overlap between Organ-Specific Proteins and Existing Host Factor Datasets

**Figure 3.**
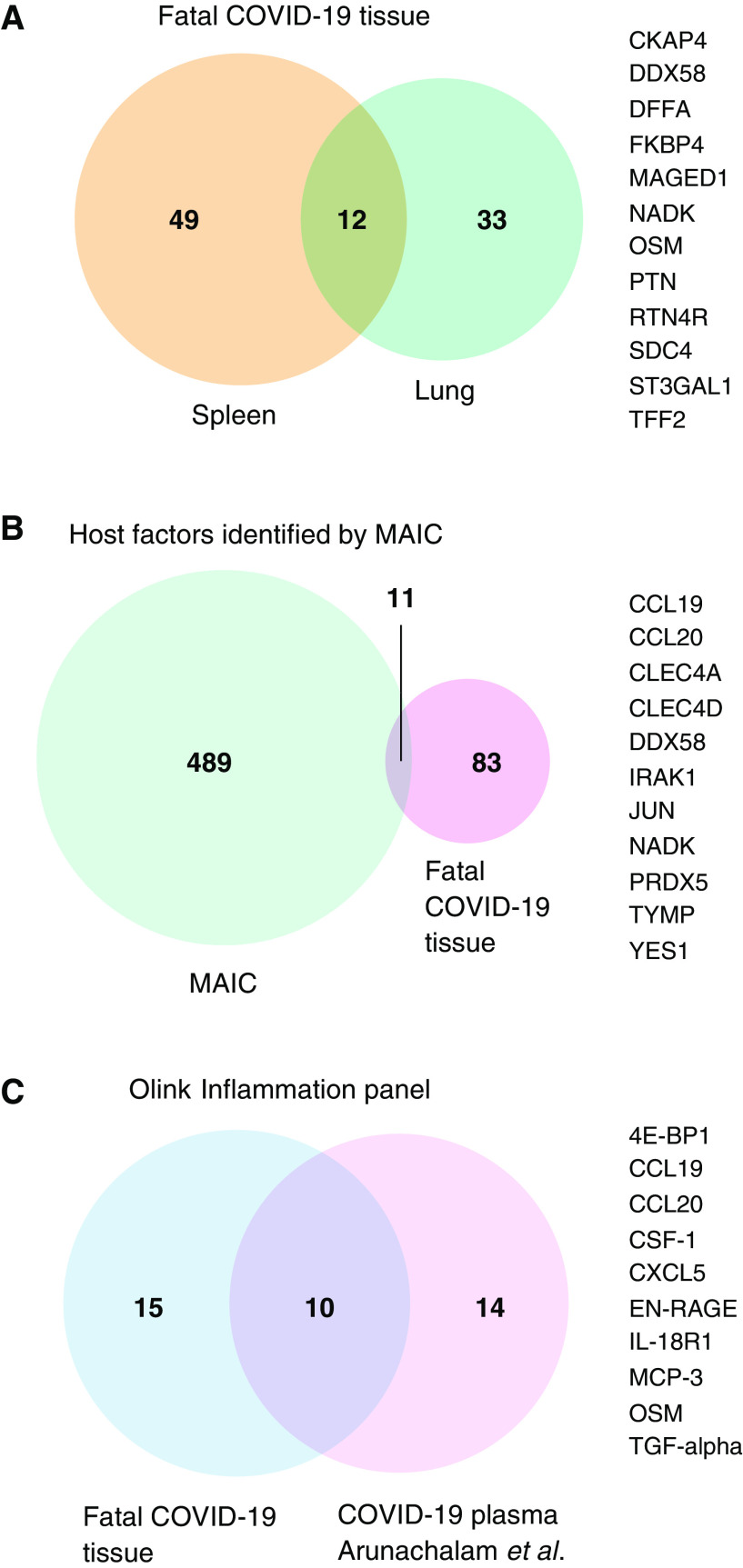
Overlap of differentially abundant tissue proteins with other datasets. Venn diagrams (*VennDiagram*; R Studio) illustrating the overlap in (*A*) differentially abundant proteins between spleen and lung tissue from this study, (*B*) differentially abundant tissue proteins from this study and the top 500 results from COVID-19 meta-analysis by information content (MAIC), and (*C*) differentially abundant tissue proteins from this study (restricted to Olink Inflammation panel) and plasma proteins reported by Arunachalam and colleagues also using the Olink Inflammation panel. Overlapping proteins are listed to the right of the Venn diagrams.

Differentially abundant proteins in lung and spleen samples from patients with fatal COVID-19 were compared, identifying 12 overlapping proteins (Figure 3A). These shared the same direction of differential abundance (increased in COVID-19 cases), with the exceptions of OSM and SDC4 (both increased in lung and decreased in spleen). Published host factors implicated in COVID-19, identified and prioritized using the meta-analysis by information content (MAIC) algorithm, were cross referenced (18). Gene lists included in the MAIC input included studies of peripheral blood and BAL fluid in humans, *in vitro* cell culture models, and animal models. We compared all differentially abundant proteins from fatal COVID-19 tissue (spleen and lung) with the top 500 ranked host factors by MAIC (as of January 2021), identifying 11 overlapping proteins (Figure 3B). Finally, we compared our differentially abundant tissue proteins with those identified as being differentially abundant in plasma of hospitalized COVID-19 patients compared with healthy controls in a previous study by Arunachalam and colleagues, using the Olink Inflammation panel (19). This identified 10 overlapping proteins (Figure 3C). The overlap with the MAIC results and differentially abundant plasma proteins enhances our confidence in the relevance of these factors in COVID-19 and also highlights that important differences exist between tissue and other body compartments (plasma and BAL fluid) and *in vitro* systems. These comparative analyses identified DDX58, OSM, TYMP, IL-18, CCL19, CCL20, MCP-3, and CSF-1 as core proteins with evidence from different sources supporting their relevance in COVID-19 immunopathology.

## Discussion

Targeted proteomic analysis of lung and spleen tissue, sites of an extensive inflammatory response in fatal COVID-19, identified differentially abundant host factors that provide several mechanistic insights into COVID-19 immunopathology specifically, and acute lung injury more broadly. Mechanisms of COVID-19 immunopathogenesis have been discovered at an unprecedented rate, including the histologic correlates of lung injury at a single-cell level (20, 21), inflammatory mediator profiles in peripheral blood (11), and the genetic basis of susceptibility to critical illness (22). Characterization of the specific inflammatory mediators present in inflamed tissues in COVID-19 is lacking but is required for the confirmation that immunopathologic processes identified through orthogonal approaches are relevant at the tissue level. This is especially relevant considering the finding that T-cell and monocyte phenotypes, and inflammatory mediator responses, differ between peripheral blood and BAL fluid in critically ill adults with COVID-19 (23).

In fatal COVID-19, there is histologic evidence of substantial monocyte/macrophage expansion within the lung parenchyma and frequent occurrence of a monocyte/myeloid cell vasculitis (1, 6), suggesting that these cells are direct contributors to the immunopathology seen in severe disease. However, the factors that promote mononuclear cell recruitment and expansion within lung tissue remain to be fully defined. Within our cohort we found that the monocyte chemotactic factor MCP-3 was increased in abundance in lung tissue in fatal COVID-19. This finding is of considerable interest given that MCP-3 is a ligand for CCR2, and high genotype-inferred pulmonary expression of CCR2 has recently been associated with risk of critical illness due to COVID-19 (22, 24). Indeed, CCR2 and MCP-3 have previously been shown to be essential for monocyte mobilization and recruitment to sites of inflammation in preclinical studies (25). Interestingly, MCP-3 is also elevated in peripheral blood in COVID-19, with higher concentrations in more severe disease, but does not appear to be elevated in either influenza virus or respiratory syncytial virus infection (19, 26).

Within our cohort we identified two clusters of COVID-19 patients based on the lung proteome (Figure 1C). Patients in the early viral cluster had a shorter illness duration before death, detectable viral RNA, and histologic evidence of less severe pulmonary inflammation. In contrast, patients in the late inflammatory cluster had a longer illness duration before death, generally lacked detectable viral RNA in the lung sample studied and had histologic evidence of more severe pulmonary inflammation. Surprisingly, MCP-3 abundance was similar in both COVID-19 clusters as well as in lung tissue with/without detectable virus (Figures E2E and E2F). This suggested that MCP-3 production was not occurring as a direct response to viral presence, providing further evidence of virus-independent immunopathology in severe disease. Alternatively, elevation could be a proximal event that occurs early in COVID-19 lung injury, there may be a failure to degrade the protein, or there may be a failure to recruit or activate cells that normally mediate MCP-3 downregulation. Circulating levels of other monocyte/macrophage chemotactic factors are elevated in blood and/or BAL fluid in COVID-19 (GM-CSF, CXCL10, and MCP-1) and positively correlate with disease severity (10, 11, 27). Although these proteins were not differentially abundant in lung tissue in our study, macrophage colony stimulating factor (M-CSF/CSF-1), a key driver of proliferation and differentiation of monocytes, macrophages, and their precursors, was increased in COVID-19 splenic tissue. As splenic monocytes can form a reservoir pool of cells that can be rapidly mobilized and recruited to sites of tissue injury, including the lung, it is possible that splenic monocytes may directly contribute to pulmonary immune cell expansion in COVID-19 (28, 29).

Pulmonary monocyte/macrophage pathology appears to be a specific feature of COVID-19. Analysis of postmortem tissue from people who died due to influenza, bacteria pneumonia, ARDS, and COVID-19 has recently shown that activated (IL-1β– and IL-6–producing) monocytes and macrophages are significantly more abundant in the COVID-19 lung (21). Similarly, at a peripheral blood level, the strong association between GM-CSF and severity in COVID-19 is not observed in influenza (in which GM-CSF levels are close to measurements in healthy control subjects) (11). Together, these results support a specific role for monocyte/macrophage immunopathology in COVID-19 and prioritize the investigation of MCP-3/CCR2 and CSF-1 signaling as therapeutic targets.

EN-RAGE/S100A12, OSM, and TYMP were increased in abundance in lung tissue with additional evidence of these factors being involved in COVID-19 provided from existing peripheral blood data sets (EN-RAGE and OSM) and MAIC (TYMP), which identifies host factors implicated in COVID-19 (18, 19). EN-RAGE is a proinflammatory myeloid cell product (especially neutrophils) and along with its receptor is increased in plasma, BAL fluid, and lung tissue in lung injury/ARDS (30, 31). In COVID-19, circulating EN-RAGE is positively associated with severity (11) and can cause activation of endothelial cells. Histologic evidence of a major role for neutrophils in COVID-19 pneumonitis is inconclusive, with patchy neutrophil-rich bronchopneumonia being a frequent but highly variable finding, although neutrophil extracellular traps and neutrophilic capillaritis in lung tissue have been reported (3, 32, 33). Peripheral blood neutrophilia and neutrophil activation occurs during severe disease (34, 35), suggesting neutrophils within the pulmonary vascular compartment may be producing EN-RAGE even in the absence of significant neutrophil recruitment to, and extravasation in, lung tissue. In addition, a sub-set of peripheral blood monocytes in severe COVID-19 highly express EN-RAGE (determined by single-cell sequencing) and could also be a relevant source (36). OSM is produced by both neutrophils and monocytes and can stimulate IL-6 production by fibroblasts and endothelial cells, in addition to increasing P-selectin expression by endothelial cells and MCP-1 expression by mesothelial cells (37–40). Circulating OSM positively correlates with COVID-19 severity, but expression by peripheral blood mononuclear cells is downregulated (19). This is consistent with our observation of increased abundance of OSM in the lung but decreased abundance in the spleen, suggesting that pulmonary tissue is a major source that may directly contribute to endothelial activation and leukocyte migration. Although not differentially abundant when comparing cases with controls, pulmonary IL-6 was lower in the late inflammatory COVID-19 patient cluster. This could indicate an early role for IL-6 in COVID-19 pathogenesis and be consistent with the clinical observation that IL-6 blockade does not shorten the duration of IMV among patients receiving IMV at initiation but does reduce risk of progression to IMV or death when initiated earlier (9). Pulmonary thrombosis is common in fatal COVID-19 and is associated with megakaryocyte expansion in the lung (1, 5, 41). TYMP was increased in abundance in lung tissue and has proangiogenic and prothrombotic functions. Whether this protein acts as a potential modifiable mediator of pulmonary thrombosis will require additional investigation (42, 43).

Splenic white pulp atrophy, and specifically CD8^+^ T-cell depletion, have been described in both fatal COVID-19 and SARS, with peripheral blood lymphopenia common and correlated with severity (1, 2, 44–46). In spleen tissue we observed decreased abundance of lymphocyte chemotactic factors and CD8A, and increased abundance of proapoptotic factors, which could contribute to lymphocyte loss. Consistent with the reactive plasmacytosis described in the spleen, proteins involved in B-cell receptor signaling were increased in abundance. IL-18R1 was also increased in splenic tissue in addition to peripheral blood in severe COVID-19 and has a role in B-cell expansion (19, 47).

### Limitations

The relatively small cohort size limited detailed comparisons between subgroups of patients and histologic characteristics. The cohort is also quite homogenous (mean age 79.6 years and 12/13 male), and the ethnicity of participants is not known, so age-, sex-, or ethnicity-related differences in immunopathologic mechanisms cannot be evaluated in this study. Control lung tissue was obtained from a cohort of people younger than the COVID-19 cohort (mean age 69 vs. 80) and comprised of more women (4/7 versus 1/13). Olink immunoassay results were not validated with an orthogonal method, although good correlation between the immunoassay results and ELISA quantification for EN-RAGE and OSM in plasma has recently been reported (19). Control lung tissue was obtained from lung cancer resections, and although only distant tissue with no histologic evidence of cancer or inflammation was used, it is possible that tissue homeostasis could have undergone subtle alterations. Similarly, control spleen samples were collected from deceased organ donors during bench surgery at the time of organ retrieval and could have undergone alterations relating to the mode of death. Furthermore, although postmortem acquisition allows analysis of lung and splenic tissue that would otherwise not be able to be acquired, measured levels of short-lived or rapidly degraded proteins may have been lower than in tissue during life. Although only a single lung lobe was sampled for proteomic analysis, and therefore could introduce sampling bias, the right middle lobe was chosen for all patients as it tended to be representative of changes in all lung lobes (viral presence, histology, and median inflammation score). Although analysis of tissue from a spectrum of COVID-19 severity would be informative, obtaining lung tissue during life from patients with COVID-19 with survivable lung injury would not be practically or ethically feasible due to the risks associated with an open lung biopsy for research purposes in critically ill patients. Similarly, while comparison of COVID-19 tissue to patients with ARDS from other etiologies is conceptually of interest (to identify factors that are truly unique to COVID-19), disease mechanisms that are shared with other causes of lung injury are also of interest as potential therapeutic targets. For example, histologic evidence of pulmonary microthrombosis is common to fatal influenza, SARS, and nonselected ARDS, in addition to COVID-19 (48), and TYMP could therefore be a factor common to these different diseases. Similarly, the evidence provided here for the role of EN-RAGE in tissue inflammation adds to the existing literature reporting its relevance in nonselected ARDS.

### Conclusions

In summary, we provide a unique perspective into tissue immunopathology in the lung parenchyma and spleen in fatal COVID-19. Importantly, our data confirm the relevance of myeloid cell immunopathology as a central pathologic process in life-threatening COVID-19 and advance the field by discovering some of the mediators of this at a protein level in inflamed tissues. Differentially abundant proteins provide mechanistic insight into previously described pathophysiological features of COVID-19, including early and late disease stages, inflammation, monocyte/macrophage recruitment, thrombosis, plasmacytosis, and splenic lymphocyte depletion, and in doing so identify potential therapeutic targets. These findings may also be relevant to ARDS owing to severe acute respiratory infections more generally.
